# The impact of oral diseases on the quality of life of patients attending the oral medicine clinic at a dental specialty centre

**DOI:** 10.1038/s41598-026-51695-5

**Published:** 2026-05-06

**Authors:** Razan A. Alenezi, Abdulhameed Alsarraf, Mahmoud K. AL-Omiri

**Affiliations:** 1 Jahra Dental Speciality Centre, Oral Medicine Clinic, Ministry of Health, Kuwait city, Kuwait; 2 Department of Fixed and Removable Prosthodontics, School of Dentistry, The University of Jordan, Amman, 11942 Jordan

**Keywords:** Oral Mucosal Diseases (OMDs), Oral Health-Related Quality of Life (OHRQoL), Oral Health Impact Profile-14 (OHIP-14), Psychosocial impact, Diseases, Health care, Medical research

## Abstract

Oral mucosal diseases (OMDs) are common and can severely affect quality of life through pain, functional limitations, and psychological distress. Their prevalence is influenced by lifestyle, socioeconomic factors, and healthcare access, making early diagnosis and effective management essential for improving patient outcomes. To assess OHRQoL in individuals affected by a variety of OMDs, examine the influence of demographic and clinical characteristics on quality-of-life outcomes, and provide insights into the factors most strongly associated with OHRQoL in this population. This cross-sectional study was conducted at an oral medicine clinic in Kuwait. Patients diagnosed with oral mucosal diseases who provided informed consent completed a paper-based, Arabic-translated version of the Oral Health Impact Profile-14 (OHIP-14) questionnaire. The survey collected demographic data and assessed the physical, psychological, and social impacts of oral disease on quality of life. Chi-square, Spearman correlation, Mann Whitney U, Kruskal Wallis, and regression tests were used to examine the associations between different variables at α = 0.05. A total of 103 patients with OMDs and 103 matched controls were included. Patients reported significantly higher psychological discomfort (*P* = .007) and psychological disability (*P* = .011) compared to controls. Females had worse Oral Health-Related Quality of Life (OHRQoL) scores than males (*P* < .05), while patients with premalignant and malignant lesions experienced the greatest impairments. Reactive lesions were associated with better OHRQoL outcomes than immune/infectious and malignant lesions (*P* < .05). OMDs significantly impair oral health-related quality of life, particularly in psychological domains, with gender and disease type emerging as key determinants. Incorporating psychosocial support into routine care is essential to enhance patient outcomes.

**Supplementary Information:**

The online version contains supplementary material available at 10.1038/s41598-026-51695-5.

## Introduction

Oral mucosal diseases (OMDs) encompass a wide range of conditions affecting the soft tissues of the oral cavity, including infections, ulcerations, potentially malignant lesions, oral cancer, and manifestations of systemic diseases^1^. These disorders can significantly impair oral health-related quality of life (OHRQoL), as they often cause physical discomfort, pain, and difficulties with daily functions such as eating, speaking, and social interaction^2–4^. Beyond the physical symptoms, patients with OMDs frequently experience psychological distress and embarrassment. When lesions are visible or interfere with function, they may also lead to social isolation and reduced self-esteem^5^.

In Kuwait, as in many parts of the world, the prevalence of oral diseases remains high. Factors such as dietary habits, oral hygiene practices, socioeconomic status, cultural attitudes, and unequal access to dental care all contribute to this burden^6,7^. Although advances in diagnosis and management have been made, many patients still experience delayed diagnosis or inadequate treatment, particularly in cases of potentially malignant disorders and oral cancer^8^. For those with advanced disease, treatment can further compromise quality of life due to the effects of surgery, radiotherapy, or chemotherapy, including facial disfigurement, speech difficulties, and swallowing impairment^9,10^.

In recent years, there has been growing recognition of the importance of evaluating quality of life in individuals with OMDs^11^. Assessing OHRQoL provides insight into the broader impact of these conditions beyond clinical signs and symptoms. Such assessments help guide treatment decisions, prioritize care, and allocate healthcare resources more effectively^12^. However, despite this increasing attention, the literature on OHRQoL in OMD patients remains limited. Much of the available research has focused on oral cancer or single disease entities, while many other OMDs remain understudied in terms of their psychosocial and functional consequences.

Improving outcomes for patients with OMDs requires a holistic approach that combines early detection, targeted prevention strategies, comprehensive clinical management, and psychosocial support. Public education on modifiable risk factors, particularly tobacco and alcohol use, is also critical in reducing the disease burden^13^. At the same time, a deeper understanding of how OMDs affect OHRQoL is essential for improving patient care and ensuring that both physical and emotional needs are addressed throughout the course of the disease.

The aim of this study was to assess OHRQoL in individuals affected by a variety of OMDs. Specifically, it sought to evaluate OHRQoL using validated assessment tools, examine the influence of demographic and clinical characteristics on quality-of-life outcomes, and provide insights into the factors most strongly associated with impaired OHRQoL in this population. The results are expected to contribute to a better understanding of the burden of OMDs, inform clinical practice, and support the development of more patient-centered approaches to care.

## Methods

This research was designed as a cross-sectional, observational, case–control study conducted at the Oral Medicine Clinic, Al-Jahra Specialty Center, Kuwait, between December 15, 2024, and September 7, 2025. The study aimed to evaluate the impact of oral mucosal diseases (OMDs) on oral health-related quality of life (OHRQoL). Ethical approval was obtained from the Kuwait Ministry of Health Institutional Review Board, Kuwait (Approval No. 2024/2690, Dated November 18, 2024). A written informed consent was obtained from the participants and/or their guardians. The ethical principles of Helsinki Declaration (9th version, 2013) were applied during this study.

A consecutive sampling approach was employed. The study group included all patients referred to the oral medicine clinic with diagnosed oral mucosal diseases during the study period who were willing and able to provide informed consent. Patients aged 18 years and older provided written informed consent themselves, while those younger than 18 participated with parental or guardian consent. The exclusion criterion was refusal to participate. In total, 103 patients were recruited.

For comparison, a control group of 103 participants matched for age and gender was recruited during the same period from the polyclinic. Controls were patients who did not have any oral mucosal disease and who attended the clinic for routine dental check-ups and dental procedures such as scaling and restorative fillings.

Data collection was conducted using a paper-based questionnaire. The first part recorded demographic and clinical details including age, gender, marital status, occupation, and clinical diagnosis. The second part used the Oral Health Impact Profile-14 (OHIP-14)^14^, a validated tool for assessing the physical, psychological, and social impact of oral conditions on quality of life. The Arabic version of the OHIP-14 was used and completed by all patients. It was previously translated and validated in the literature^15^, and has been applied in several other studies assessing oral health-related quality of life^16,17^. The OHIP-14 consists of 14 items, each rated on a 5-point Likert scale (0 = never to 4 = very often), with total scores ranging from 0 to 56. Higher scores indicate a greater negative impact on daily life. The OHIP-14 includes the following 7 dimensions: functional limitations (items 1 and 2), physical pain (items 3 and 4), psychological discomfort (items 5 and 6), physical disability (items 7 and 8), psychological disability (items 9 and 10), social disability (items 11 and 12), and handicap (items 13 and 14). Functional limitation, discomfort and pain related dimensions measure physiological, biological or immediate impacts due to discomfort, pain, or difficulties in word pronunciation^14–17^. Meanwhile, disability related dimensions (psychological, physical, or social) evaluate specific intermediate impacts on daily life and consequences of limitations, for example having to avoid certain food, interrupt meals, or facing difficulties carrying out usual daily activities^14–17^. Meanwhile, handicap dimension assesses the cumulative effects of disabilities on the life of individual as well as the largest impacts on overall quality of life, for example perceiving life as less satisfactory in general or being unable to function^14–17^. The OHIP-14 is based on Locker’s theoretical model of progression of impacts on quality of life where impressions (e.g. pain) could lead to functional limitations that might lead to disability, which in turn might end up in handicap. The OHIP-14 dimensions and items are included in the Supplementary Table. All participants completed the questionnaire during their clinic visit, after providing informed consent.

### Statistical analysis

Data analysis in this study was performed using the SPSS computer software (IBM SPSS Statistics v21.0; IBM Corp., USA). A two tailed α of 0.05 was used to identify the significance of the statistical results. The Shapiro-Wilk test was used to test normal distribution of the continuous data and revealed that all continuous variables (age and OHIP total, dimension, and item scores) in this study were not normally distributed. Consequently, the descriptive statistics for the continuous variables included the mean, standard deviation, median, interquartile range, minimum value, and maximum values. Meanwhile, frequencies and percentages were used to describe the categorical variables in this study (gender, marital status, occupation, and diagnosis).

Chi square test was used to find associations between group and each of gender, marital status, and occupation among the study sample. The Mann Whitney U test was used to recognize differences in OHIP scores between genders, marital status, and patient and control groups. The Kruskal Wallis test was used to discover variation in OHIP scores between different categories of diagnoses among the patients as well as between occupations. Further pairwise comparisons were conducted using Mann Whitney U test to identify differences in OHIP scores between each pair of diagnoses as well as between each pair of occupations. Correlations between age and OHIP scores were identified using the Spearman’s Rho test. Multiple linear regression analyses were conducted to predict the OHIP scores and reveal the contribution of having oral lesion or not and the category of diagnosis towards the of OHIP scores considering the confounding effects of gender, marital status, occupation, and age. The residuals normal distribution was tested via the P-P and scatter plots before conducting the linear regression.

A priori power analysis using the G*Power computer program (version 3.1.9.7; Heinrich-Heine University) estimated a minimum sample size of 92 participants. The estimation utilized a linear multiple regression test (F test family) with a significance level (α) of 0.05, an effect size of 0.3, a statistic power (1 – β) of 0.8, and 5 predictors. The OHIP scores were used as the primary outcome measure for this purpose. To guarantee the estimated sample size throughout this investigation, more participants (extra 18 participants) were recruited to compensate for potential participant drop outs. Accordingly, one hundred and five participants were recruited for this investigation.

## Results

This investigation included 105 participants (42 males and 63 females) who were diagnosed with oral disease or condition. Two male participants did not complete the required OHIP test (drop out ratio was 1.9%). Consequently, 103 participants (40 males and 63 females) were included in this investigation, provided all the requested data, and had their data analyzed. Patients’ mean age was 43.0 years old (SD = 18.0, Median = 42.0, IQR = 24), and ranged between 17 and 84 years old. In addition, the investigation included 103 control participants who matched the patients group by age and gender. Controls’ mean age was 42.3 years old (SD = 15.3, Median = 41.0, IQR = 20), and ranged between 17 and 84 years old.

Table 1 presents the descriptive statistics for categorical variables including gender, marital status, occupation, and category of diagnosis of oral condition among the study sample. More patients were married and unemployed than controls (*P*= .007 and 0.002 respectively, Table 1).

**Table 1:** Distribution of categorical variables among the study participants and controls (*N* = 103 patients and 103 controls).

Categorical variables		Patients (*N* = 103)		Controls (*N* = 103)		Comparison between patients and controls			
		N	%	N	%	X^2^	df	P	ES
Gender	Male	40	38.8	40	38.8	0.000	1	1.000	Phi = 0.000
	Female	63	61.2	63	61.2				
Marital status	Single	32	31.1	52	50.5	8.041	1	0.007	Phi = 0.198
	Married	71	68.9	51	49.5				
Occupation	Unemployed	48	46.6	23	22.3	14.576	3	0.002	Cramer’s V = 0.266
	Student	18	17.5	22	21.4				
	Private sector	12	11.7	24	23.3				
	Government	25	24.3	34	33.0				
Category of diagnosis	Developmental	11	10.7	–	–	–			
	Reactive	44	42.7	–	–				
	Immune and infection	30	29.1	–	–				
	Malignant and premalignant	18	17.5	–	–				

X^2^= Chi square test, df= Degree of freedom, P = Two-tailed probability value, ES= Effect size.

Table 2 demonstrates the descriptive statistics and group variations in OHIP scores among the study sample. The patients reported higher psychological discomfort and psychological disability than the controls (Effect size *r* = .070 and 0.064 respectively, *P*< .05, Table 2). Specifically, patients felt more tense (item 6) and found it more difficult to relax (item 9) because of their oral condition or disease (*r* = .090 and 0.121 respectively, *P*< .05, Table 2).

**Table 2:** Descriptive statistics and group variations in OHIP scores among the study sample (*N* = 103 patients and 103 controls).

OHIP scores	Group	Descriptive statistics						Group comparisons		
		Mean	SD	Med	IQR	Min	Max	MWU	Z	P
Functional limitation	Patients	2.07	2.478	2.00	4	0	8	4865.0	-1.067	0.286
	Controls	2.23	2.197	2.00	4	0	8			
Physical pain	Patients	4.25	2.886	4.00	5	0	8	5155.0	− 0.354	0.724
	Controls	4.42	2.749	6.00	4	0	8			
Psychological discomfort	Patients	4.15	2.632	4.00	4	0	8	4172.0	-2.683	0.007
	Controls	3.16	2.527	2.00	3	0	8			
Physical disability	Patients	3.10	2.949	2.00	5	0	8	4951.0	− 0.840	0.401
	Controls	3.29	2.550	3.00	5	0	8			
Psychological disability	Patients	4.67	2.495	4.00	4	0	8	4220.5	-2.558	0.011
	Controls	3.72	2.610	4.00	4	0	8			
Social disability	Patients	2.84	2.761	2.00	5	0	8	5219.0	− 0.204	0.838
	Controls	2.67	2.467	2.00	4	0	8			
Handicap	Patients	2.76	2.771	2.00	5	0	8	5185.5	− 0.285	0.776
	Controls	2.73	2.426	2.00	4	0	8			
OHIP total score	Patients	23.83	14.240	24.00	20	0	56	5045.5	− 0.606	0.545
	Controls	22.21	15.032	22.00	24	0	55			
Q1. Trouble pronouncing any words?	Patients	0.96	1.335	0.00	2	0	4	4758.0	-1.369	0.171
	Controls	1.06	1.178	1.00	1	0	4			
Q2. Sense of taste has worsened?	Patients	1.11	1.407	0.00	2	0	4	4847.0	-1.131	0.258
	Controls	1.17	1.216	1.00	2	0	4			
Q3. Had painful aching in mouth?	Patients	2.29	1.538	3.00	3	0	4	5056.5	− 0.595	0.552
	Controls	2.21	1.432	3.00	2	0	4			
Q4. Uncomfortable to eat any foods?	Patients	1.96	1.633	2.00	4	0	4	4925.5	− 0.909	0.363
	Controls	2.20	1.471	3.00	2	0	4			
Q5. Self conscious because of mouth?	Patients	1.83	1.615	1.00	4	0	4	4770.0	-1.285	0.199
	Controls	1.49	1.335	1.00	3	0	4			
Q6. Felt tense because of mouth?	Patients	2.31	1.566	2.00	3	0	4	4033.5	-3.041	0.002
	Controls	1.67	1.396	1.00	3	0	4			
Q7. Diet been unsatisfactory?	Patients	1.46	1.513	1.00	2	0	4	4838.5	-1.123	0.261
	Controls	1.60	1.353	1.00	3	0	4			
Q8. Interrupt meals because of mouth?	Patients	1.64	1.602	1.00	3	0	4	5145.5	− 0.383	0.702
	Controls	1.69	1.449	1.00	3	0	4			
Q9. Difficult to relax because of mouth?	Patients	2.84	1.384	3.00	2	0	4	3847.5	-3.532	< 0.001
	Controls	2.17	1.496	3.00	2	0	4			
Q10. Embarrassed because of mouth?	Patients	1.83	1.611	2.00	4	0	4	4882.0	-1.014	0.311
	Controls	1.55	1.348	1.00	3	0	4			
Q11. Irritable with other people because of mouth?	Patients	1.47	1.558	1.00	3	0	4	5287.0	− 0.043	0.966
	Controls	1.36	1.327	1.00	2	0	4			
Q12. Difficulty doing usual jobs because of mouth?	Patients	1.38	1.476	1.00	3	0	4	5274.0	− 0.074	0.941
	Controls	1.31	1.299	1.00	2	0	4			
Q13. Life in general was less satisfying because of mouth?	Patients	1.49	1.527	1.00	3	0	4	5264.0	− 0.098	0.922
	Controls	1.37	1.321	1.00	2	0	4			
Q14. Totally unable to function because of mouth?	Patients	1.27	1.429	1.00	2	0	4	4872.0	-1.052	0.293
	Controls	1.36	1.220	1.00	2	0	4			

OHIP= Oral Health Impact Profile – 14, SD= Standard deviation, Med= Median, IQR= Interquartile range, Min= Minimum value, Max= Maximum value, MWU= Mann Whitney U test statistic, Z = Z statistic, P = Two-tailed probability value.

Table 3 presents gender based descriptive statistics and variations in OHIP scores among the study sample. Females had higher OHIP item, dimension, and total scores (i.e. worse oral health impacts) than males (*P*< .05), except that they reported similar self-consciousness (item 5) and irritability with other people (item 11) because of their oral condition or disease (*P*> .05, Table 3).

**Table 3:** Gender based descriptive statistics and variations in OHIP scores among the study sample (*N* = 80 males and 126 females).

OHIP scores	Gender	Descriptive statistics						Gender comparisons		
		Mean	SD	Med	IQR	Min	Max	MWU	Z	P
Functional limitation	Male	1.36	1.693	0.00	2	0	7	3601.5	-3.424	0.001
	Female	2.62	2.541	2.00	4	0	8			
Physical pain	Male	3.44	2.812	3.00	6	0	8	3545.5	-3.474	0.001
	Female	4.87	2.685	6.00	4	0	8			
Psychological discomfort	Male	3.18	2.604	2.00	5	0	8	4147.0	-2.006	0.045
	Female	3.93	2.602	4.00	4	0	8			
Physical disability	Male	2.42	2.510	2.00	4	0	8	3691.0	-3.131	0.002
	Female	3.66	2.794	4.00	5	0	8			
Psychological disability	Male	3.51	2.542	4.00	4	0	8	3755.0	-2.955	0.003
	Female	4.60	2.542	5.00	4	0	8			
Social disability	Male	2.18	2.229	2.00	4	0	8	4073.0	-2.203	0.028
	Female	3.10	2.769	2.00	6	0	8			
Handicap	Male	2.05	2.182	2.00	4	0	7	3833.5	-2.802	0.005
	Female	3.16	2.743	2.00	6	0	8			
OHIP total score	Male	18.14	12.465	16.00	18	0	44	3464.0	-3.632	< 0.001
	Female	25.94	15.085	27.00	22	0	56			
Q1. Trouble pronouncing any words?	Male	0.68	1.006	0.00	1	0	4	3866.5	-2.848	0.004
	Female	1.21	1.350	1.00	2	0	4			
Q2. Sense of taste has worsened?	Male	0.69	0.977	0.00	1	0	4	3540.0	-3.645	< 0.001
	Female	1.41	1.412	1.00	3	0	4			
Q3. Had painful aching in mouth?	Male	1.90	1.527	2.00	3	0	4	3908.5	-2.624	0.009
	Female	2.47	1.420	3.00	3	0	4			
Q4. Uncomfortable to eat any foods?	Male	1.55	1.518	1.00	3	0	4	3469.5	-3.711	< 0.001
	Female	2.40	1.492	3.00	3	0	4			
Q5. Self conscious because of mouth?	Male	1.49	1.492	1.00	3	0	4	4421.5	-1.354	0.176
	Female	1.76	1.483	1.00	3	0	4			
Q6. Felt tense because of mouth?	Male	1.69	1.507	1.00	3	0	4	4063.5	-2.232	0.026
	Female	2.17	1.495	2.00	3	0	4			
Q7. Diet been unsatisfactory?	Male	1.18	1.275	1.00	2	0	4	3943.0	-2.549	0.011
	Female	1.74	1.487	1.00	3	0	4			
Q8. Interrupt meals because of mouth?	Male	1.23	1.423	1.00	2	0	4	3691.5	-3.175	0.001
	Female	1.92	1.529	2.00	2	0	4			
Q9. Difficult to relax because of mouth?	Male	2.19	1.522	3.00	3	0	4	4050.5	-2.295	0.022
	Female	2.69	1.424	3.00	3	0	4			
Q10. Embarrassed because of mouth?	Male	1.31	1.407	1.00	2	0	4	3810.0	-2.868	0.004
	Female	1.91	1.495	2.00	2	0	4			
Q11. Irritable with other people because of mouth?	Male	1.25	1.349	1.00	2	0	4	4475.0	-1.234	0.217
	Female	1.51	1.495	1.00	3	0	4			
Q12. Difficulty doing usual jobs because of mouth?	Male	0.94	1.174	1.00	1	0	4	3676.0	-3.247	0.001
	Female	1.59	1.450	1.00	3	0	4			
Q13. Life in general was less satisfying because of mouth?	Male	1.12	1.267	1.00	2	0	4	4016.5	-2.380	0.017
	Female	1.61	1.486	1.00	3	0	4			
Q14. Totally unable to function because of mouth?	Male	0.94	1.092	1.00	1	0	4	3790.0	-2.958	0.003
	Female	1.54	1.403	1.00	3	0	4			

OHIP= Oral Health Impact Profile – 14, SD= Standard deviation, Med= Median, IQR= Interquartile range, Min= Minimum value, Max= Maximum value, MWU= Mann Whitney U test statistic, Z = Z statistic, P = Two-tailed probability value.

Considering marital status, no differences in any OHIP item, dimension, or total scores were found between single or married participants (*P*> .05). Based on occupation, no differences in any OHIP item, dimension, or total scores were found between participants (*P*> .05), except for functional limitations (X^23^= 11.045, *P*= .011, Effect size η²= 0.040), trouble pronouncing words (item 1; X^23^= 12.923, *P*= .005, η²= 0.049), and sense of taste has worsened (item 2; X^23^= 7.900, *P*= .048, η²= 0.024). Further pairwise comparisons between occupation pairs showed that unemployed participants had less functional limitations (MWU test statistic = 903.5, *P*= .010, Effect size *r* = .062), less trouble pronouncing words (item 1; MWU = 902.5, *P*= .007, *r* = .067), and less sense of taste worsening (item 2, MWU = 969.0, *P*= .029, *r* = .044) than the participants who worked in private sector. In addition, unemployed participants had less functional limitations (MWU = 1031.0, *P*= .013, *r* = .056), less trouble pronouncing words (item 1; MWU = 1010.5, *P*= .007, *r* = .066), and less sense of taste worsening (item 2, MWU = 1065.0, *P*= .020, *r* = .048) than the students.

Table 4 presents the descriptive statistics and differences in OHIP item, dimension, and total scores based on the category of diagnosis of oral condition among the patients group. Physical disability dimension, handicap dimension, worsened sense of taste (item 2), interrupted meals (item 8), and totally unable to function (item 14) were significantly different between the categories of diagnosis (*P*< .05, Table 4). Further pairwise comparisons between each two categories of diagnosis showed that reactive lesions were associated with less sense of taste worsening (item 2) and better ability to function (item 14) than developmental and pigmentation lesions (*P*< .05, Table 5). Also, reactive lesions were associated with less functional limitation, physical disability, handicap, sense of taste worsening (item 2), interrupted meals (item 8), difficulty doing usual jobs (item 12), inability to function (item 14) than immune and infectious lesions (*P*< .05, Table 5). Furthermore, reactive lesions were associated with less physical pain, physical disability, social disability, handicap, OHIP total score, uncomfortable food eating (item 4), unsatisfactory diet (item 7), interrupted meals (item 8), irritability with other people (item 11), dissatisfaction with life in general (item 13), and inability to function (item 14) than premalignant and malignant lesions (*P*< .05, Table 5). Finally, premalignant and malignant lesions were associated with more irritability with other people (item 11) than immune and infectious lesions (*P*= .046, Table 5).

**Table 4:** Descriptive statistics and variations in OHIP scores based on category of oral condition diagnosis among the patients (*N* = 103 patients).

OHIP scores	Diagnosis Category*	Descriptive statistics						Group comparisons		
		Mean	SD	Med	IQR	Min	Max	X^2^ (df = 3)	P	η²
Functional limitation	1	2.55	2.423	2.00	5	0	6	5.783	0.123	0.028
	2	1.41	1.834	0.00	3	0	7			
	3	2.90	2.893	2.00	5	0	8			
	4	2.00	2.849	0.50	3	0	8			
Physical pain	1	3.45	2.979	3.00	5	0	8	7.541	0.057	0.046
	2	3.59	2.679	4.00	6	0	8			
	3	4.83	2.878	6.00	5	0	8			
	4	5.39	2.993	7.00	5	0	8			
Psychological discomfort	1	3.64	2.803	4.00	4	0	8	0.386	0.943	0.026
	2	4.18	2.563	4.00	4	0	8			
	3	4.17	2.705	4.00	5	0	8			
	4	4.33	2.765	3.50	5	0	8			
Physical disability	1	2.55	2.339	2.00	3	0	8	8.562	0.036	0.056
	2	2.14	2.358	2.00	4	0	8			
	3	4.03	3.275	4.50	7	0	8			
	4	4.22	3.335	4.50	8	0	8			
Psychological disability	1	4.36	2.730	5.00	4	0	8	0.039	0.998	0.030
	2	4.70	2.474	4.00	3	0	8			
	3	4.70	2.493	4.50	4	0	8			
	4	4.72	2.608	4.50	4	0	8			
Social disability	1	3.64	2.838	4.00	5	0	8	6.864	0.076	0.039
	2	2.11	2.470	1.00	4	0	8			
	3	3.00	2.901	2.00	5	0	8			
	4	3.89	2.867	4.00	5	0	8			
Handicap	1	3.55	2.945	3.00	6	0	8	8.274	0.041	0.053
	2	1.86	2.349	1.00	3	0	8			
	3	3.20	2.987	2.00	6	0	8			
	4	3.72	2.824	4.50	6	0	8			
OHIP total score	1	23.73	14.759	24.00	17	0	47	5.432	0.143	0.025
	2	20.00	11.291	17.50	17	0	43			
	3	26.83	17.066	26.00	33	0	53			
	4	28.28	13.940	26.50	20	2	56			
Q1. Trouble pronouncing any words?	1	0.91	0.944	1.00	2	0	2	2.155	0.541	0.009
	2	0.75	1.164	0.00	1	0	4			
	3	1.30	1.579	0.50	3	0	4			
	4	0.94	1.474	0.00	1	0	4			
Q2. Sense of taste has worsened?	1	1.64	1.629	1.00	3	0	4	8.403	0.038	0.055
	2	0.66	0.987	0.00	1	0	3			
	3	1.60	1.589	1.00	3	0	4			
	4	1.06	1.552	0.00	2	0	4			
Q3. Had painful aching in mouth?	1	1.82	1.662	1.00	4	0	4	5.203	0.158	0.022
	2	2.02	1.532	2.00	3	0	4			
	3	2.60	1.476	3.00	2	0	4			
	4	2.72	1.487	3.50	2	0	4			
Q4. Uncomfortable to eat any foods?	1	1.64	1.629	1.00	4	0	4	7.584	0.055	0.046
	2	1.57	1.576	1.00	3	0	4			
	3	2.23	1.633	3.00	3	0	4			
	4	2.67	1.572	3.50	3	0	4			
Q5. Self conscious because of mouth?	1	1.73	1.679	1.00	4	0	4	1.481	0.687	0.015
	2	1.86	1.679	2.00	4	0	4			
	3	1.63	1.608	1.00	4	0	4			
	4	2.17	1.505	2.00	3	0	4			
Q6. Felt tense because of mouth?	1	1.91	1.578	1.00	3	0	4	1.224	0.747	0.018
	2	2.32	1.611	2.50	3	0	4			
	3	2.53	1.456	3.00	3	0	4			
	4	2.17	1.689	2.00	3	0	4			
Q7. Diet been unsatisfactory?	1	1.45	1.440	1.00	2	0	4	5.316	0.150	0.023
	2	1.02	1.210	1.00	2	0	4			
	3	1.80	1.710	1.50	4	0	4			
	4	1.94	1.697	1.50	4	0	4			
Q8. Interrupt meals because of mouth?	1	1.09	1.221	1.00	2	0	4	10.089	0.018	0.072
	2	1.11	1.298	1.00	2	0	4			
	3	2.23	1.716	3.00	4	0	4			
	4	2.28	1.809	3.00	4	0	4			
Q9. Difficult to relax because of mouth?	1	2.64	1.629	3.00	3	0	4	0.294	0.961	0.027
	2	2.89	1.368	3.00	2	0	4			
	3	2.83	1.341	3.00	2	0	4			
	4	2.89	1.451	3.50	2	0	4			
Q10. Embarrassed because of mouth?	1	1.73	1.489	1.00	2	0	4	0.021	0.999	0.030
	2	1.82	1.674	2.00	4	0	4			
	3	1.87	1.613	1.50	3	0	4			
	4	1.83	1.654	1.50	3	0	4			
Q11. Irritable with other people because of mouth?	1	2.00	1.673	2.00	4	0	4	6.846	0.077	0.039
	2	1.20	1.549	0.00	3	0	4			
	3	1.23	1.455	1.00	2	0	4			
	4	2.17	1.505	2.50	3	0	4			
Q12. Difficulty doing usual jobs because of mouth?	1	1.64	1.433	1.00	3	0	4	7.040	0.071	0.041
	2	0.91	1.197	0.50	1	0	4			
	3	1.77	1.612	1.00	4	0	4			
	4	1.72	1.674	1.50	3	0	4			
Q13. Life in general was less satisfying because of mouth?	1	1.91	1.578	2.00	3	0	4	6.792	0.079	0.038
	2	1.05	1.363	0.00	2	0	4			
	3	1.67	1.539	1.00	3	0	4			
	4	2.00	1.680	2.00	4	0	4			
Q14. Totally unable to function because of mouth?	1	1.64	1.433	1.00	3	0	4	9.581	0.022	0.066
	2	0.82	1.225	0.00	1	0	4			
	3	1.53	1.548	1.00	3	0	4			
	4	1.72	1.487	1.50	4	0	4			

*Categories of diagnosis: 1 = Developmental conditions, 2 = Reactive lesions, 3 = Immune and infectious lesions, 4 = Malignant and Premalignant lesions. OHIP= Oral Health Impact Profile – 14, SD= Standard deviation, Med= Median, IQR= Interquartile range, Min= Minimum value, Max= Maximum value, X^2^= Chi square statistic utilizing the Kruskal Wallis test, df= Degree of freedom, P = Two-tailed probability value, η²= Eta squared effect size.

**Table 5:** Differences in OHIP scores between different categories of oral condition diagnosis among the patients (*N* = 103 patients).

Variable	Mann Whitney U test between each 2 categories of diagnosis						
	Statistics	*Compared categories of diagnosis					
		1 and 2	1 and 3	1 and 4	2 and 3	2 and 4	3 and 4
Functional limitations	MWU (P)	177.0 (0.140)	157.5 (0.821)	82.5 (0.435)	465.5 (**0.024**)	374.0 (0.710)	215.0 (0.224)
	r	0.199	0.035	0.145	0.263 485.5 (0.052)	0.047	0.175
Physical pain	MWU (P)	232.0 (0.831)	120.0 (0.180)	63.5 (0.099)		255.5 (**0.027**)	226.5 (0.343)
	r	0.029	0.209	0.306	0.226	0.280	0.137
Psychological discomfort	MWU (P)	216.0 (0.577)	147.5 (0.603)	86.0 (0.555)	659.5 (0.996)	396.0 (1.000)	260.5 (0.838)
	r	0.075	0.081	0.110	0.001	0.000	0.029
Physical disability	MWU (P)	209.0 (0.473)	127.5 (0.262)	72.0 (0.216)	442.5 (**0.014**)	255.0 (**0.024**)	265.0 (0.913)
	r	0.097	0.175	0.230	0.285	0.287	0.016
Psychological disability	MWU (P)	237.5 (0.923)	158.0 (0.835)	94.0 (0.820)	650.0 (0.911)	395.0 (0.987)	267.5 (0.957)
	r	0.013	0.032	0.042	0.013	0.002	0.008
Social disability	MWU (P)	162.0 (0.082)	144.0 (0.531)	95.0 (0.856)	531.0 (0.145)	252.5 (**0.022**)	223.5 (0.317)
	r	0.234	0.098	0.034	0.169	0.291	0.145
Handicap	MWU (P)	161.0 (0.074)	156.5 (0.798)	98.5 (0.982)	472.0 (**0.032**)	252.5 (**0.020**)	242.0 (0.545)
	r	0.241	0.040	0.004	0.249	0.295	0.087
OHIP total score	MWU (P)	193.0 (0.302)	145.0 (0.556)	83.0 (0.472)	513.0 (0.105)	260.5 (**0.036**)	252.5 (0.709)
	r	0.139	0.092	0.134	0.188	0.267	0.054
Q1. Trouble pronouncing any words?	MWU (P)	206.5 (0.402)	153.5 (0.717)	88.0 (0.591)	546.0 (0.163)	373.0 (0.686)	242.5 (0.525)
	r	0.113	0.057	0.100	0.162	0.051	0.092
Q2. Sense of taste has worsened?	MWU (P)	157.0 (**0.047**)	163.5 (0.964)	78.0 (0.313)	436.5 (**0.008**)	357.0 (0.493)	214.0 (0.209)
	r	0.268	0.007	0.188	0.310	0.087	0.181
Q3. Had painful aching in mouth?	MWU (P)	229.0 (0.779)	123.0 (0.202)	67.0 (0.133)	514.5 (0.099)	286.5 (0.081)	251.5 (0.680)
	r	0.038	0.199	0.279	0.192	0.221	0.059
Q4. Uncomfortable to eat any foods?	MWU (P)	228.5 (0.768)	136.5 (0.387)	66.0 (0.119)	503.5 (0.075)	239.5 (**0.012**)	223.0 (0.297)
	r	0.040	0.135	0.290	0.207	0.318	0.151
Q5. Self conscious because of mouth?	MWU (P)	236.0 (0.896)	155.0 (0.761)	82.5 (0.446)	608.5 (0.557)	351.0 (0.472)	214.0 (0.221)
	r	0.018	0.048	0.141	0.068	0.091	0.177
Q6. Felt tense because of mouth?	MWU (P)	209.0 (0.472)	128.0 (0.262)	91.0 (0.709)	619.5 (0.644)	379.0 (0.784)	240.5 (0.515)
	r	0.097	0.175	0.069	0.054	0.035	0.094
Q7. Diet been unsatisfactory?	MWU (P)	197.0 (0.318)	153.5 (0.726)	85.5 (0.530)	505.5 (0.075)	275.0 (**0.050**)	253.5 (0.715)
	r	0.135	0.055	0.116	0.207	0.249	0.053
Q8. Interrupt meals because of mouth?	MWU (P)	236.0 (0.894)	108.5 (0.086)	66.0 (0.123)	426.0 (**0.008**)	256.5 (**0.024**)	260.5 (0.832)
	r	0.018	0.268	0.286	0.311	0.287	0.031
Q9. Difficult to relax because of mouth?	MWU (P)	225.0 (0.703)	160.5 (0.889)	91.0 (0.701)	629.5 (0.722)	390.5 (0.927)	253.0 (0.701)
	r	0.051	0.022	0.071	0.041	0.012	0.055
Q10. Embarrassed because of mouth?	MWU (P)	241.0 (0.983)	162.0 (0.928)	98.5 (0.982)	648.5 (0.896)	395.5 (0.994)	265.0 (0.913)
	r	0.003	0.014	0.004	0.015	0.001	0.016
Q11. Irritable with other people because of mouth?	MWU (P)	173.5 (0.125)	121.5 (0.182)	94.5 (0.836)	628.5 (0.710)	261.5 (**0.028**)	179.5 (**0.046**)
	r	0.207	0.208	0.038	0.043	0.279	0.287
Q12. Difficulty doing usual jobs because of mouth?	MWU (P)	168.0 (0.098)	157.0 (0.808)	98.0 (0.963)	455.5 (**0.018**)	296.0 (0.099)	256.5 (0.766)
	r	0.223	0.038	0.009	0.275	0.210	0.043
Q13. Life in general was less satisfying because of mouth?	MWU (P)	164.0 (0.081)	152.0 (0.694)	96.0 (0.890)	496.0 (0.058)	271.5 (**0.040**)	243.5 (0.562)
	r	0.235	0.061	0.026	0.221	0.260	0.084
Q14. Totally unable to function because of mouth?	MWU (P)	156.0 (**0.048**)	158.0 (0.831)	96.0 (0.890)	455.0 (**0.016**)	249.5 (**0.014**)	249.5 (0.652)
	r	0.266	0.033	0.026	0.281	0.311	0.065

*Categories: 1 = Developmental conditions, 2 = Reactive lesions, 3 = Immune and infectious lesions, 4 = Malignant and Premalignant lesions. OHIP= Oral Health Impact Profile – 14, MWU= Mann Whitney U test statistic, P = Two-tailed probability value, r= Effect size r.

Correlations between age and OHIP item, dimension, and total scores showed no significant correlations between age and any OHIP scores (*P*> .05), except that older participants were more tense because of their mouth (item 6, *P*= .049, Spearman Rho = 0.137) than younger participants.

Multiple linear regression analyses (Table 6 and Supplementary Table) were conducted to identify the predictive potentials and contributions of having oral condition or not and the category of the diagnosis of oral condition towards the OHIP item, dimension, and total scores while taking into account the confounding effects of gender, age, marital status, and occupation.

**Table 6:** Linear regression analyses to predict OHIP scores utilizing the demographic variables, having oral condition or not, and category of oral condition diagnosis among the study sample (*N* = 206; 103 patients and 103 controls).

Dependent variable	Predictors*	UnSC		SC	t	P	95% CI for B	
		B	SE	Beta			Lower Bound	Upper Bound
Functional limitations R^2^= 0.158 DW = 1.525	Constant	-1.799	1.179	–	-1.526	0.129	-4.124	0.526
	Gender	1.171	0.332	0.243	3.525	0.001	0.516	1.825
	Immune Category^$^	1.231	0.542	0.186	2.274	0.024	0.163	2.299
	Student Occupation^#^	1.324	0.619	0.225	2.140	0.034	0.104	2.544
	Private Occupation^#^	1.608	0.501	0.262	3.212	0.002	0.621	2.596
Physical pain R^2^= 0.134 DW = 1.574	Constant	-1.243	1.438	–	− 0.864	0.389	-4.080	1.594
	Gender	1.413	0.405	0.244	3.488	0.001	0.614	2.212
	Malignant Category^$^	1.955	0.786	0.197	2.487	0.014	0.404	3.505
Psychological discomfort R^2^= 0.070 DW = 2.069	Constant	3.000	1.389	–	2.160	0.032	0.261	5.739
	Gender	0.800	0.391	0.148	2.045	0.042	0.028	1.572
	Group	-1.152	0.484	− 0.220	-2.378	0.018	-2.107	− 0.197
Physical disability R^2^= 0.114 DW = 1.794	Constant	-1.431	1.423	–	-1.006	0.316	-4.237	1.375
	Gender	1.228	0.401	0.216	3.064	0.002	0.438	2.018
	Group	1.033	0.496	0.188	2.082	0.039	0.055	2.011
	Immune Category^$^	1.557	0.654	0.200	2.382	0.018	0.268	2.846
	Malignant Category^$^	2.331	0.778	0.240	2.998	0.003	0.797	3.864
Psychological disability R^2^= 0.087 DW = 1.721	Constant	3.531	1.360	–	2.596	0.010	0.849	6.213
	Gender	1.205	0.383	0.226	3.147	0.002	0.450	1.961
	Group	-1.065	0.474	− 0.206	-2.246	0.026	-2.000	− 0.130
Social disability R^2^= 0.085 DW = 1.887	Constant	0.979	1.373	–	0.713	0.477	-1.729	3.687
	Gender	1.133	0.387	0.210	2.928	0.004	0.370	1.896
	Malignant Category^$^	2.202	0.750	0.239	2.934	0.004	0.722	3.682
Handicap R^2^= 0.119 DW = 1.961	Constant	0.569	1.340	–	0.425	0.671	-2.073	3.212
	Gender	1.262	0.377	0.236	3.344	0.001	0.518	2.006
	Developmental Category^$^	1.756	0.847	0.152	2.074	0.039	0.086	3.426
	Malignant Category^$^	1.979	0.732	0.216	2.703	0.007	0.535	3.423
OHIP total score R^2^= 0.110 DW = 1.687	Constant	3.606	7.581	–	0.476	0.635	-11.345	18.558
	Gender	8.212	2.135	0.272	3.845	< 0.001	4.000	12.423
	Malignant Category^$^	9.915	4.143	0.192	2.393	0.018	1.744	18.085
Q1. Trouble pronouncing any words? R^2^= 0.121 DW = 1.575	Constant	− 0.732	0.648	–	-1.130	0.260	-2.009	0.546
	Gender	0.495	0.182	0.191	2.714	0.007	0.135	0.855
	Student Occupation^#^	0.746	0.340	0.235	2.194	0.029	0.075	1.416
	Private Occupation^#^	0.896	0.275	0.271	3.257	0.001	0.354	1.439
Q2. Sense of taste has worsened? R^2^= 0.110 DW = 1.640	Constant	-1.067	0.663	–	-1.608	0.109	-2.375	0.241
	Gender	0.675	0.187	0.250	3.614	< 0.001	0.307	1.044
	Developmental Category^$^	1.015	0.419	0.174	2.420	0.016	0.188	1.842
	Immune Category^$^	0.789	0.305	0.213	2.590	0.010	0.188	1.390
	Private Occupation^#^	0.712	0.282	0.207	2.527	0.012	0.156	1.268
Q3. Had painful aching in mouth? R^2^= 0.090 DW = 1.528	Constant	− 0.115	0.777	–	− 0.148	0.883	-1.648	1.418
	Gender	0.534	0.219	0.175	2.439	0.016	0.102	0.966
Q4. Uncomfortable to eat any foods? R^2^= 0.111 DW = 1.741	Constant	-1.128	0.786	–	-1.436	0.153	-2.678	0.421
	Gender	0.879	0.221	0.274	3.973	< 0.001	0.443	1.316
	Group	0.583	0.274	0.188	2.128	0.035	0.043	1.123
	Malignant Category^$^	1.194	0.429	0.217	2.782	0.006	0.348	2.041
Q6. Felt tense because of mouth? R^2^= 0.090 DW = 1.947	Constant	1.672	0.794	–	2.107	0.036	0.107	3.238
	Gender	0.441	0.224	0.141	1.972	0.050	0.000	0.882
	Group	− 0.719	0.277	− 0.238	-2.598	0.010	-1.265	− 0.173
Q7. Diet been unsatisfactory? R^2^= 0.086 DW = 1.811	Constant	− 0.499	0.753	–	− 0.663	0.508	-1.984	0.985
	Gender	0.553	0.212	0.187	2.608	0.010	0.135	0.971
	Group	0.537	0.263	0.188	2.045	0.042	0.019	1.055
	Malignant Category^$^	1.082	0.411	0.214	2.629	0.009	0.270	1.893
Q8. Interrupt meals because of mouth? R^2^= 0.125 DW = 1.900	Constant	− 0.932	0.783	–	-1.190	0.236	-2.476	0.612
	Gender	0.675	0.221	0.215	3.061	0.003	0.240	1.110
	Immune Category^$^	0.887	0.360	0.206	2.467	0.014	0.178	1.596
	Malignant Category^$^	1.249	0.428	0.232	2.919	0.004	0.405	2.093
Q9. Difficult to relax because of mouth? R^2^= 0.090 DW = 1.713	Constant	2.806	0.774	–	3.625	< 0.001	1.280	4.333
	Gender	0.554	0.218	0.182	2.542	0.012	0.124	0.984
	Group	− 0.729	0.270	− 0.247	-2.699	0.008	-1.261	− 0.196
Q10. Embarrassed because of mouth? R^2^= 0.067 DW = 1.829	Constant	0.725	0.790	–	0.917	0.360	− 0.834	2.283
	Gender	0.651	0.223	0.212	2.925	0.004	0.212	1.090
Q11. Irritable with other people because of mouth? R^2^= 0.073 DW = 1.818	Constant	0.703	0.764	–	0.920	0.359	− 0.804	2.211
	Malignant Category^$^	1.165	0.418	0.228	2.788	0.006	0.341	1.989
Q12. Difficulty doing usual jobs because of mouth? R^2^= 0.114 DW = 1.962	Constant	0.276	0.718	–	0.384	0.701	-1.139	1.691
	Gender	0.715	0.202	0.250	3.537	0.001	0.316	1.113
	Immune Category^$^	0.670	0.330	0.171	2.032	0.043	0.020	1.320
	Malignant Category^$^	1.037	0.392	0.212	2.645	0.009	0.264	1.810
Q13. Life in general was less satisfying because of mouth? R^2^= 0.106 DW = 2.103	Constant	0.775	0.740	–	1.047	0.296	− 0.685	2.235
	Gender	0.597	0.209	0.203	2.861	0.005	0.185	1.008
	Malignant Category^$^	1.016	0.405	0.202	2.512	0.013	0.218	1.814
Q14. Totally unable to function because of mouth? R^2^= 0.114 DW = 1.869	Constant	− 0.206	0.686	–	− 0.300	0.764	-1.558	1.146
	Gender	0.666	0.193	0.243	3.446	0.001	0.285	1.046
	Developmental Category^$^	0.863	0.433	0.147	1.992	0.048	0.008	1.718
	Malignant Category^$^	0.963	0.375	0.206	2.570	0.011	0.224	1.702

R^2^= Coefficient of determination, DW= Durbin Watson statistic, UnSC= Unstandardized coefficient, SC= Standardized coefficient, B= Beta statistics, SE= Standard Error, t = t statistic, P = Two-tailed probability value, CI= Confidence intervals. *Gender, age, group (have condition versus controls), marital status, diagnosis categories, and occupation categories were entered in the regression model, and only significant predictors are presented in the table. ^$^Reference category is reactive lesions category. ^#^Reference occupation is unemployed category.

The regression analyses showed that having an oral condition was associated with worse physical oral health impacts since it was associated with higher physical disability (*P*= .039, B = 1.033), uncomfortable food eating (item 4, *P*= .035, B = 0.583), and unsatisfactory diet (item 7, *P*= .042, B = 0.537) in comparison to not having oral condition. Meanwhile, having an oral condition was associated with better psychological oral health impacts since it was associated with lower psychological discomfort (*P*= .018, B= -1.152), psychological disability (*P*= .026, B= -1.065), feeling tense because of mouth (item 6, *P*= .010, B= -0.719), and difficulty to relax (item 9, *P*= .008, B= -0.729) in comparison to not having oral condition.

The regression analyses (Table 6 and Supplementary Table) also showed that reactive lesions were associated with better oral health impacts since they were associated with less functional limitations (*P*= .024, B = 1.231), physical disability (*P*= .018, B = 1.557), sense of taste worsening (item 2, *P*= .010, B = 0.789), interrupted meals (item 8, *P*= .014, B = 0.887), and difficulty doing usual jobs (item 12, *P*= .043, B = 0.670) in comparison to immune and infectious lesions. Furthermore, reactive lesions were associated with less physical pain (*P*= .014, B = 1.955), physical disability (*P*= .003, B = 2.331), social disability (*P*= .004, B = 2.202, handicap (*P*= .007, B = 1.979), total OHIP scores (*P*= .018, B = 9.915, uncomfortable food eating (item 4, *P*= .006, B = 1.194), unsatisfactory diet (item item 7, *P*= .009, B = 1.082), interrupted meals (item 8, *P*= .004, B = 1.249), irritability with other people (item 11, *P*= .006, B = 1.165), difficulty doing usual jobs (item 12, *P*= .009, B = 1.037), dissatisfaction with life in general (item 13, *P*= .013, B = 1.061), and inability to function (item 14, *P*= .011, B = 0.963) in comparison to premalignant and malignant lesions. Moreover, reactive lesions were associated with less handicap (*P*= .039, B = 1.756), sense of taste worsening (item 2, *P*= .016, B = 1.015), and inability to function (item 14, *P*= .048, B = 0.863) in comparison to developmental lesions.

The regression analyses (Table 6 and Supplementary Table) also revealed that being a female was associated with worse oral health impacts as females scored higher on all OHIP items, dimensions, and total scores than males (*P*< .05), except for being self conscious (item 5) and feeling irritable with other people (item 11). Besides, being unemployed was associated with better oral health impacts as they were associated with less functional limitations (*P*= .002, B = 1.608), trouble pronouncing any words (item 1, *P*= .001, B = 0.896), and sense of taste worsening (item 2, *P*= .012, B = 0.712) in comparison to private occupation. Also, being unemployed was associated with less functional limitations (*P*= .034, B = 1.324) and less trouble pronouncing any words (item 1, *P*= .029, B = 0.746) in comparison to students.

## Discussion

The null hypothesis of this study was that there would be no difference in oral health impact profile (OHIP-14) scores between patients with oral mucosal diseases (OMDs) and controls, and no difference between the various categories of OMDs. Our results showed that patients with OMDs experienced significantly worse oral health-related quality of life (OHRQoL) compared to controls, and that the type of oral condition had a substantial influence on the level of impairment. Therefore, the null hypothesis was rejected.

This study identified that patients with oral mucosal diseases had worse OHRQoL, particularly in psychological domains such as tension and difficulty relaxing. These findings agree with previous studies that demonstrated the considerable psychosocial burden associated with OMDs, where patients frequently reported embarrassment, reduced self-esteem, and social withdrawal in addition to pain and functional limitations^18^. Research on oral lichen planus, recurrent aphthous stomatitis, candidiasis, and oral cancer has consistently reported similar impairments in daily functioning and emotional wellbeing, which supports the outcomes observed in our sample^19–21^. Interestingly, in some adjusted regression models, patients with oral mucosal diseases demonstrated slightly better psychological outcomes than controls. This unexpected finding may reflect adaptive coping mechanisms developed by patients living with chronic oral conditions or the reassurance provided by regular clinical follow-up and professional support.

Differences were also observed between disease categories. Patients with premalignant and malignant lesions reported the greatest reductions in OHRQoL, in line with earlier studies that highlighted the profound impacts of oral cancer and potentially malignant disorders due to speech difficulties, disfigurement, and treatment-related side effects^22,23^. In contrast, patients with reactive lesions had the least impairment, which agrees with earlier reports that benign conditions generally have a milder impact on quality of life compared to immune-mediated or malignant diseases^24^.

Gender also played an important role, as females consistently reported worse OHRQoL scores than males. This finding is consistent with previous literature^25,26^, and may reflect differences in health perception, psychosocial coping, and cultural expectations. Furthermore, employment status appeared to influence patients’ experiences as unemployed individuals reported fewer negative oral health impacts than students or those working in the private sector. Although there is limited evidence on the association between occupational status and OHRQoL, this observation suggests that psychosocial and lifestyle factors may shape how patients perceive the burden of oral disease^27^.

This study has several limitations. The male-to-female ratio was not equal, which reflects the natural flow of patients attending the Oral Medicine Clinic between December 15, 2024 and September 7, 2025. Another limitation is that data collection relied on self-reported questionnaires, which are subject to patient perception and recall bias. However, the OHIP-14 is a well-validated, reliable, and widely used tool^28^, which strengthens the robustness of the results. In addition, the cross-sectional design and the single-center setting in this investigation might limit the causal inference and generalizability of the study outcomes. Finally, the number of patients attending the oral medicine clinic was limited, which restricted the overall sample size. Although the investigators aimed to achieve a larger sample, the relatively low flow of patients to the oral medicine clinic during the study period made this beyond author control.

Future studies are recommended to incorporate psychological and personality assessments to provide a more comprehensive understanding of the relationship between psychosocial factors and OHRQoL. Multicenter research with larger, more diverse populations representing different races and cultural backgrounds would also help to strengthen the generalizability of the findings. In addition, longitudinal studies are advised to evaluate how OHRQoL changes over time and in response to treatment^29,30^.

## Conclusion

OMDs substantially impair oral health-related quality of life, particularly in psychological domains. Gender and disease type are major determinants of impact, with women and patients with premalignant or malignant lesions being the most affected. These findings highlight the need for comprehensive, patient-centered approaches that integrate clinical management with psychosocial support and preventive strategies to improve outcomes and overall wellbeing.

**Tables**.

## Supplementary Information

Below is the link to the electronic supplementary material.

Supplementary Material 1

## Data Availability

The datasets generated and analyzed during the current study are available from the corresponding author on reasonable request.

## Abbreviations

OMDs: Oral mucosal diseases
OHRQoL: Oral health related quality of life
OHIP-14: Oral Health Impact Profile 14
