# Supplementary material for: The impact of oral diseases on the quality of life of patients attending the oral medicine clinic at a dental specialty centre
Source: Sci Rep. 2026 May 6;16:20952. doi: 10.1038/s41598-026-51695-5 (PMC13338210; doi:10.1038/s41598-026-51695-5)
Supplement: Supplementary file 1 — Supplementary Material 1 [file 41598_2026_51695_MOESM1_ESM.pdf]

Supplementary material

Supplementary Table. Detailed linear regression analyses to predict OHIP scores utilizing the demographic variables, having oral condition or not, and category of oral condition diagnosis among the study sample (N= 206; 103 patients and 103 controls).

Dependent variable	Predictors*	Unst Co		St Co	t	P	95% CI for B	
		B	SE	Beta			Lower Bound	Upper Bound
Functional limitations	Constant	-1.799	1.179	---	-1.526	.129	-4.124	.526
	Gender	1.171	.332	.243	3.525	.001	.516	1.825
	Age	.014	.015	.105	.965	.336	-.015	.043
R^2= .158	Group	.605	.411	.130	1.472	.143	-.205	1.416
DW=1.525	Marital Status	-.212	.442	-.045	-.480	.632	-1.083	.659
	Developmental Category$	1.219	.745	.118	1.636	.103	-.250	2.689
	Immune Category$	1.231	.542	.186	2.274	.024	.163	2.299
	Malignant Category$	.821	.644	.099	1.274	.204	-.450	2.091
	Student Occupation#	1.324	.619	.225	2.140	.034	.104	2.544
	Private Occupation#	1.608	.501	.262	3.212	.002	.621	2.596
	Government Occupation#	.330	.451	.064	.731	.466	-.560	1.219
Physical pain	Constant	-1.243	1.438	---	-.864	.389	-4.080	1.594
	Gender	1.413	.405	.244	3.488	.001	.614	2.212
R^2= .134	Age	.002	.018	.011	.100	.920	-.033	.037
DW=1.574	Group	.785	.502	.140	1.565	.119	-.204	1.774
	Marital Status	.902	.539	.158	1.674	.096	-.160	1.965
	Developmental Category$	-.013	.909	-.001	-.014	.989	-1.805	1.780
	Immune Category$	.819	.661	.103	1.240	.216	-.484	2.122
	Malignant Category$	1.955	.786	.197	2.487	.014	.404	3.505
	Student Occupation#	1.202	.755	.170	1.593	.113	-.286	2.691
	Private Occupation#	.670	.611	.091	1.096	.274	-.535	1.875
	Government Occupation#	-.142	.550	-.023	-.259	.796	-1.227	.943
Psychological discomfort	Constant	3.000	1.389	---	2.160	.032	.261	5.739
	Gender	.800	.391	.148	2.045	.042	.028	1.572
	Age	.003	.017	.020	.178	.859	-.031	.037
R^2= .070	Group	-1.152	.484	-.220	-2.378	.018	-2.107	-.197
DW=2.069	Marital Status	.412	.520	.077	.791	.430	-.614	1.438
	Developmental Category$	-.565	.878	-.049	-.644	.520	-2.296	1.166
	Immune Category$	-.329	.638	-.044	-.516	.607	-1.587	.929
	Malignant Category$	.223	.759	.024	.293	.770	-1.274	1.720
	Student Occupation#	.626	.729	.095	.859	.391	-.811	2.064
	Private Occupation#	.547	.590	.079	.927	.355	-.617	1.710
	Government Occupation#	.520	.531	.090	.979	.329	-.528	1.568
Physical disability	Constant	-1.431	1.423	---	-1.006	.316	-4.237	1.375
	Gender	1.228	.401	.216	3.064	.002	.438	2.018
R^2= .114	Age	.005	.018	.035	.312	.755	-.029	.040

DW=1.794	Group	1.033	.496	.188	2.082	.039	.055	2.011
	Marital Status	.088	.533	.016	.164	.870	-.964	1.139
	Developmental Category[$]	.464	.899	.038	.517	.606	-1.309	2.238
	Immune Category[$]	1.557	.654	.200	2.382	.018	.268	2.846
	Malignant Category[$]	2.331	.778	.240	2.998	.003	.797	3.864
	Student Occupation[#]	.672	.747	.097	.900	.369	-.801	2.145
	Private Occupation[#]	.644	.604	.089	1.065	.288	-.548	1.836
	Government Occupation[#]	.123	.544	.020	.225	.822	-.951	1.196
Psychological	Constant	3.531	1.360	---	2.596	.010	.849	6.213
disability	Gender	1.205	.383	.226	3.147	.002	.450	1.961
	Age	.006	.017	.040	.352	.726	-.027	.039
R^2= .087	Group	-1.065	.474	-.206	-2.246	.026	-2.000	-.130
DW=1.721	Marital Status	-.082	.509	-.016	-.162	.872	-1.087	.922
	Developmental Category[$]	-.351	.859	-.031	-.408	.683	-2.046	1.344
	Immune Category[$]	-.302	.625	-.041	-.483	.630	-1.534	.930
	Malignant Category[$]	.405	.743	.044	.545	.587	-1.061	1.870
	Student Occupation[#]	.604	.714	.092	.846	.398	-.804	2.012
	Private Occupation[#]	.175	.578	.026	.303	.762	-.964	1.315
	Government Occupation[#]	.315	.520	.055	.605	.546	-.711	1.340
Social disability	Constant	.979	1.373	---	.713	.477	-1.729	3.687
	Gender	1.133	.387	.210	2.928	.004	.370	1.896
R^2= .085	Age	.000	.017	.002	.017	.986	-.033	.034
DW=1.887	Group	.504	.479	.097	1.052	.294	-.441	1.448
	Marital Status	-.613	.514	-.116	-1.192	.235	-1.628	.401
	Developmental Category[$]	1.597	.868	.138	1.840	.067	-.115	3.308
	Immune Category[$]	.676	.631	.091	1.072	.285	-.568	1.920
	Malignant Category[$]	2.202	.750	.239	2.934	.004	.722	3.682
	Student Occupation[#]	-.353	.721	-.054	-.490	.625	-1.774	1.069
	Private Occupation[#]	-.196	.583	-.029	-.336	.738	-1.346	.955
	Government Occupation[#]	-.442	.525	-.077	-.841	.401	-1.478	.594
Handicap	Constant	.569	1.340	---	.425	.671	-2.073	3.212
	Gender	1.262	.377	.236	3.344	.001	.518	2.006
R^2= .119	Age	-.009	.016	-.064	-.576	.566	-.042	.023
DW=1.961	Group	.594	.467	.115	1.271	.205	-.328	1.515
	Marital Status	-.326	.502	-.062	-.649	.517	-1.316	.664
	Developmental Category[$]	1.756	.847	.152	2.074	.039	.086	3.426
	Immune Category[$]	.843	.616	.115	1.370	.172	-.371	2.057
	Malignant Category[$]	1.979	.732	.216	2.703	.007	.535	3.423
	Student Occupation[#]	-1.298	.703	-.198	-1.846	.066	-2.685	.089
	Private Occupation[#]	.220	.569	.032	.386	.700	-.903	1.342
	Government Occupation[#]	-.244	.513	-.043	-.477	.634	-1.255	.766
OHIP total score	Constant	3.606	7.581	---	.476	.635	-11.345	18.558
	Gender	8.212	2.135	.272	3.845	<.001	4.000	12.423
R^2= .110	Age	.021	.093	.025	.225	.822	-.163	.205
DW=1.687	Group	1.304	2.643	.045	.493	.622	-3.910	6.517

	Marital Status	.168	2.840	.006	.059	.953	-5.433	5.769
	Developmental Category$	4.108	4.791	.063	.857	.392	-5.342	13.557
	Immune Category$	4.496	3.482	.109	1.291	.198	-2.372	11.364
	Malignant Category$	9.915	4.143	.192	2.393	.018	1.744	18.085
	Student Occupation#	2.778	3.979	.075	.698	.486	-5.069	10.625
	Private Occupation#	3.668	3.220	.095	1.139	.256	-2.683	10.019
	Government Occupation#	.458	2.900	.014	.158	.875	-5.261	6.177
Q1. Trouble pronouncing any words? R^2= .121 DW=1.575	Constant	-.732	.648	---	-1.130	.260	-2.009	.546
	Gender	.495	.182	.191	2.714	.007	.135	.855
	Age	.008	.008	.119	1.066	.288	-.007	.024
	Group	.207	.226	.082	.916	.361	-.239	.652
	Marital Status	-.087	.243	-.034	-.359	.720	-.566	.391
	Developmental Category$	.204	.409	.037	.499	.618	-.603	1.012
	Immune Category$	.442	.298	.124	1.486	.139	-.145	1.029
	Malignant Category$	.265	.354	.060	.748	.455	-.433	.963
	Student Occupation#	.746	.340	.235	2.194	.029	.075	1.416
	Private Occupation#	.896	.275	.271	3.257	.001	.354	1.439
	Government Occupation#	.158	.248	.057	.640	.523	-.330	.647
Q2. Sense of taste has worsened? R^2= .110 DW=1.640	Constant	-1.067	.663	---	-1.608	.109	-2.375	.241
	Gender	.675	.187	.250	3.614	<.001	.307	1.044
	Age	.006	.008	.074	.675	.501	-.011	.022
	Group	.398	.231	.152	1.722	.087	-.058	.855
	Marital Status	-.125	.249	-.047	-.503	.616	-.615	.365
	Developmental Category$	1.015	.419	.174	2.420	.016	.188	1.842
	Immune Category$	.789	.305	.213	2.590	.010	.188	1.390
	Malignant Category$	.556	.363	.120	1.533	.127	-.159	1.271
	Student Occupation#	.578	.348	.175	1.661	.098	-.108	1.265
	Private Occupation#	.712	.282	.207	2.527	.012	.156	1.268
	Government Occupation#	.171	.254	.059	.675	.501	-.329	.672
Q3. Had painful aching in mouth? R^2= .090 DW=1.528	Constant	-.115	.777	---	-.148	.883	-1.648	1.418
	Gender	.534	.219	.175	2.439	.016	.102	.966
	Age	.004	.010	.043	.377	.707	-.015	.022
	Group	.202	.271	.068	.745	.457	-.333	.736
	Marital Status	.457	.291	.152	1.569	.118	-.117	1.031
	Developmental Category$	-.176	.491	-.027	-.357	.721	-1.144	.793
	Immune Category$	.416	.357	.099	1.166	.245	-.288	1.120
	Malignant Category$	.760	.425	.145	1.790	.075	-.078	1.598
	Student Occupation#	.756	.408	.202	1.854	.065	-.048	1.561
	Private Occupation#	.277	.330	.071	.838	.403	-.375	.928
	Government Occupation#	.070	.297	.021	.236	.814	-.516	.657
Q4. Uncomfortable to eat any foods? R^2= .111 DW=1.741	Constant	-1.128	.786	---	-1.436	.153	-2.678	.421
	Gender	.879	.221	.274	3.973	<.001	.443	1.316
	Age	-.002	.010	-.021	-.189	.850	-.021	.017
	Group	.583	.274	.188	2.128	.035	.043	1.123
	Marital Status	.445	.294	.141	1.513	.132	-.135	1.026

	Developmental Category$	.163	.497	.024	.328	.743	-.816	1.142
	Immune Category$	.403	.361	.092	1.117	.265	-.309	1.115
	Malignant Category$	1.194	.429	.217	2.782	.006	.348	2.041
	Student Occupation#	.446	.412	.114	1.082	.281	-.367	1.259
	Private Occupation#	.393	.334	.096	1.178	.240	-.265	1.051
	Government Occupation#	-.212	.300	-.062	-.707	.480	-.805	.380
Q5. Self conscious because of mouth?<br><br>R^2= .045<br>DW=2.112	Constant	1.328	.799	---	1.661	.098	-.249	2.904
	Gender	.359	.225	.117	1.595	.112	-.085	.803
	Age	.005	.010	.060	.520	.604	-.014	.025
	Group	-.433	.279	-.146	-1.553	.122	-.983	.117
	Marital Status	-.017	.299	-.006	-.056	.955	-.607	.574
	Developmental Category$	-.159	.505	-.024	-.314	.754	-1.155	.838
	Immune Category$	-.319	.367	-.076	-.869	.386	-1.043	.405
	Malignant Category$	.414	.437	.079	.948	.344	-.447	1.276
	Student Occupation#	.504	.420	.134	1.200	.232	-.324	1.331
	Private Occupation#	.331	.340	.085	.975	.331	-.338	1.001
	Government Occupation#	.333	.306	.101	1.089	.278	-.270	.936
Q6. Felt tense because of mouth?<br><br>R^2= .090<br>DW=1.947	Constant	1.672	.794	---	2.107	.036	.107	3.238
	Gender	.441	.224	.141	1.972	.050	.000	.882
	Age	-.002	.010	-.024	-.213	.832	-.021	.017
	Group	-.719	.277	-.238	-2.598	.010	-1.265	-.173
	Marital Status	.429	.297	.139	1.441	.151	-.158	1.015
	Developmental Category$	-.407	.502	-.061	-.811	.418	-1.396	.583
	Immune Category$	-.010	.365	-.002	-.027	.978	-.729	.709
	Malignant Category$	-.192	.434	-.036	-.442	.659	-1.047	.664
	Student Occupation#	.123	.417	.032	.295	.769	-.699	.944
	Private Occupation#	.216	.337	.054	.640	.523	-.449	.881
	Government Occupation#	.187	.304	.056	.616	.538	-.412	.786
Q7. Diet been unsatisfactory?<br><br>R^2= .086<br>DW=1.811	Constant	-.499	.753	---	-.663	.508	-1.984	.985
	Gender	.553	.212	.187	2.608	.010	.135	.971
	Age	-.004	.009	-.051	-.453	.651	-.022	.014
	Group	.537	.263	.188	2.045	.042	.019	1.055
	Marital Status	.148	.282	.051	.525	.600	-.408	.704
	Developmental Category$	.492	.476	.077	1.035	.302	-.446	1.431
	Immune Category$	.670	.346	.165	1.937	.054	-.012	1.352
	Malignant Category$	1.082	.411	.214	2.629	.009	.270	1.893
	Student Occupation#	.227	.395	.063	.573	.567	-.553	1.006
	Private Occupation#	.147	.320	.039	.460	.646	-.484	.778
	Government Occupation#	-.147	.288	-.047	-.512	.609	-.715	.421
Q8. Interrupt meals because of mouth?<br><br>R^2= .125<br>DW=1.900	Constant	-.932	.783	---	-1.190	.236	-2.476	.612
	Gender	.675	.221	.215	3.061	.003	.240	1.110
	Age	.010	.010	.111	1.004	.317	-.009	.029
	Group	.496	.273	.163	1.818	.071	-.042	1.035
	Marital Status	-.061	.293	-.020	-.207	.836	-.639	.518
	Developmental Category$	-.028	.495	-.004	-.056	.955	-1.004	.948

	Immune Category$	.887	.360	.206	2.467	.014	.178	1.596
	Malignant Category$	1.249	.428	.232	2.919	.004	.405	2.093
	Student Occupation#	.445	.411	.116	1.084	.280	-.365	1.256
	Private Occupation#	.497	.333	.124	1.493	.137	-.159	1.152
	Government Occupation#	.270	.299	.080	.901	.369	-.321	.860
Q9. Difficult to relax because of mouth? R^2= .090 DW=1.713	Constant	2.806	.774	---	3.625	<.001	1.280	4.333
	Gender	.554	.218	.182	2.542	.012	.124	.984
	Age	-.004	.010	-.048	-.424	.672	-.023	.015
	Group	-.729	.270	-.247	-2.699	.008	-1.261	-.196
	Marital Status	.080	.290	.027	.275	.784	-.492	.652
	Developmental Category$	-.235	.489	-.036	-.481	.631	-1.200	.730
	Immune Category$	-.178	.356	-.043	-.501	.617	-.880	.523
	Malignant Category$	.208	.423	.040	.493	.623	-.626	1.043
	Student Occupation#	-.002	.406	-.001	-.005	.996	-.803	.799
	Private Occupation#	-.257	.329	-.066	-.782	.435	-.906	.391
	Government Occupation#	-.067	.296	-.021	-.226	.821	-.651	.517
Q10. Embarrassed because of mouth? R^2= .067 DW=1.829	Constant	.725	.790	---	.917	.360	-.834	2.283
	Gender	.651	.223	.212	2.925	.004	.212	1.090
	Age	.010	.010	.117	1.021	.309	-.009	.029
	Group	-.337	.275	-.113	-1.222	.223	-.880	.207
	Marital Status	-.162	.296	-.054	-.548	.585	-.746	.422
	Developmental Category$	-.116	.499	-.018	-.232	.817	-1.100	.869
	Immune Category$	-.123	.363	-.029	-.340	.735	-.839	.593
	Malignant Category$	.196	.432	.037	.455	.650	-.655	1.048
	Student Occupation#	.606	.415	.161	1.462	.145	-.212	1.424
	Private Occupation#	.432	.336	.111	1.289	.199	-.229	1.094
	Government Occupation#	.381	.302	.116	1.262	.208	-.215	.977
Q11. Irritable with other people because of mouth? R^2= .073 DW=1.818	Constant	.703	.764	---	.920	.359	-.804	2.211
	Gender	.418	.215	.140	1.940	.054	-.007	.842
	Age	-.001	.009	-.008	-.071	.943	-.019	.018
	Group	.122	.267	.042	.459	.647	-.403	.648
	Marital Status	-.186	.286	-.063	-.648	.518	-.750	.379
	Developmental Category$	.844	.483	.132	1.747	.082	-.109	1.797
	Immune Category$	.006	.351	.002	.018	.986	-.686	.699
	Malignant Category$	1.165	.418	.228	2.788	.006	.341	1.989
	Student Occupation#	.191	.401	.052	.476	.635	-.600	.982
	Private Occupation#	.141	.325	.037	.435	.664	-.499	.782
	Government Occupation#	-.148	.292	-.046	-.505	.614	-.724	.429
Q12. Difficulty doing usual jobs because of mouth? R^2= .114 DW=1.962	Constant	.276	.718	---	.384	.701	-1.139	1.691
	Gender	.715	.202	.250	3.537	.001	.316	1.113
	Age	.001	.009	.012	.108	.914	-.016	.018
	Group	.381	.250	.138	1.524	.129	-.112	.875
	Marital Status	-.428	.269	-.152	-1.591	.113	-.958	.102
	Developmental Category$	.753	.453	.122	1.660	.098	-.141	1.647
	Immune Category$	.670	.330	.171	2.032	.043	.020	1.320

	Malignant Category$	1.037	.392	.212	2.645	.009	.264	1.810
	Student Occupation#	-.544	.377	-.155	-1.444	.150	-1.286	.199
	Private Occupation#	-.337	.305	-.092	-1.105	.270	-.938	.264
	Government Occupation#	-.294	.274	-.096	-1.072	.285	-.835	.247
Q13. Life in general was less satisfying because of mouth?	Constant	.775	.740	---	1.047	.296	-.685	2.235
	Gender	.597	.209	.203	2.861	.005	.185	1.008
	Age	-.005	.009	-.064	-.574	.567	-.023	.013
	Group	.152	.258	.053	.588	.557	-.357	.661
	Marital Status	-.266	.277	-.092	-.959	.339	-.813	.281
R^2= .106	Developmental Category$	.893	.468	.141	1.908	.058	-.030	1.816
DW=2.103	Immune Category$	.385	.340	.096	1.132	.259	-.286	1.056
	Malignant Category$	1.016	.405	.202	2.512	.013	.218	1.814
	Student Occupation#	-.724	.389	-.202	-1.865	.064	-1.491	.042
	Private Occupation#	.201	.314	.054	.640	.523	-.419	.821
	Government Occupation#	-.043	.283	-.014	-.152	.879	-.602	.515
Q14. Totally unable to function because of mouth?	Constant	-.206	.686	---	-.300	.764	-1.558	1.146
	Gender	.666	.193	.243	3.446	.001	.285	1.046
	Age	-.004	.008	-.056	-.505	.614	-.021	.012
	Group	.442	.239	.167	1.849	.066	-.029	.914
R^2= .114	Marital Status	-.060	.257	-.022	-.232	.817	-.566	.447
DW=1.869	Developmental Category$	.863	.433	.147	1.992	.048	.008	1.718
	Immune Category$	.458	.315	.122	1.455	.147	-.163	1.079
	Malignant Category$	.963	.375	.206	2.570	.011	.224	1.702
	Student Occupation#	-.573	.360	-.171	-1.594	.113	-1.283	.136
	Private Occupation#	.019	.291	.005	.064	.949	-.556	.593
	Government Occupation#	-.201	.262	-.069	-.768	.444	-.719	.316

R^2= Coefficient of determination, DW= Durbin Watson statistic, Unst Co= Unstandardized coefficient, St Co= Standardized coefficient, B= Beta statistics, SE= Standard Error, t= t statistic, P= Two-tailed probability value, CI= Confidence intervals. $Reference category is reactive lesions category. #Reference occupation is unemployed category.
